# *N*-Alkyl Carbazole Derivatives as New Tools for Alzheimer’s Disease: Preliminary Studies

**DOI:** 10.3390/molecules19079307

**Published:** 2014-07-02

**Authors:** Carmela Saturnino, Domenico Iacopetta, Maria Stefania Sinicropi, Camillo Rosano, Anna Caruso, Angelamaria Caporale, Nancy Marra, Barbara Marengo, Maria Adelaide Pronzato, Ortensia Ilaria Parisi, Pasquale Longo, Roberta Ricciarelli

**Affiliations:** 1 Department of Pharmacy, University of Salerno, via Giovanni Paolo II, 132, Fisciano 84084 (SA), Italy; E-Mails: saturnino@unisa.it (C.S.); acaporale@unisa.it (A.C.); nancyma@hotmail.it (N.M.); 2 Department of Pharmacy, Health and Nutritional Sciences, University of Calabria, Arcavacata di Rende 87036 (CS), Italy; E-Mails: domenico.iacopetta@unical.it (D.I.); anna.caruso@unical.it (A.C.); ortensiailaria.parisi@unical.it (O.I.P.); 3 Biopolymers and Proteomics IRCCS AOU San Martino-IST National Institute for Cancer Research Largo Benzi 10, Genova 16132, Italy; E-Mail: camillo.rosano@gmail.com; 4 Department of Computer Engineering, Modeling, Electronics and Systems, University of Calabria, Rende 87036 (CS), Italy; 5 Department of Experimental Medicine, Section of General Pathology, University of Genova, via L.B. Alberti 2, Genova 16132, Italy; E-Mails: barbara.marengo@unige.it (B.M.); maidep@unige.it (M.A.P.); ricciarelli@medicina.unige.it (R.R.); 6 Department of Sciences, University of Salerno, via Giovanni Paolo II, 132, 84084 Fisciano (SA), Italy

**Keywords:** *N*-alkyl carbazole derivatives, Alzheimer’s disease, amyloid β-peptide

## Abstract

Alzheimer’s disease (AD) is a progressive and age-related neurodegenerative disorder affecting brain cells and is the most common form of “dementia”, because of the cognitive detriment which takes place. Neuronal disruption represents its major feature, due to the cytosolic accumulation of amyloid β-peptide (Aβ) which leads to senile plaques formation and intracellular neurofibrillary tangles. Many studies have focused on the design and therapeutic use of new molecules able to inhibit Aβ aggregation. In this context, we evaluated the ability of two recently synthesized series of *N*-alkyl carbazole derivatives to increase the Aβ soluble forms, through molecular docking simulations and *in vitro* experiments. Our data evidenced that two carbazole derivatives, the most active, adopt distinct binding modes involving key residues for Aβ fibrillization. They exhibit a good interfering activity on Aβ aggregation in mouse (N2a) cells, stably expressing wild-type human amyloid precursor protein (APP) 695. These preliminary results are promising and we are confident that the *N*-alkyl carbazole derivatives may encourage next future studies needed for enlarging the knowledge about the AD disease approach.

## 1. Introduction

Alzheimer’s disease (AD) is an age-related disorder that affects brain cells, leads to the inability to conduct a normal life and because of the chronic and progressive cognitive detriment [1,2]. The neuropathological hallmarks of AD include abundant deposits of amyloid β (Aβ) peptides organized in senile plaques, accumulation of hyperphosphorylated tau protein in neurofibrillary tangles, and extensive neuronal degeneration and loss.

Aβ is a small self-aggregating molecule deriving from proteolytic processing of the amyloid precursor protein (APP). Sequential cleavage of APP by β- and γ-secretases gives rise to Aβ [3,4], which takes two prevalent forms in humans, Aβ 40 and Aβ 42, although longer and shorter peptides also exist.

The increased extracellular accumulation of Aβ variants, particularly Aβ 42, leads to the formation of Aβ fibrils, the major constituents of cerebral amyloid plaques, with subsequently neurodegeneration and brain atrophy [5].

Indeed, several pathogenic mechanisms have been proposed to explain the neurotoxicity of Aβ, including the Aβ-induced stimulation of nitric oxide (NO) production or the enhancement of glutamate neurotoxicity NMDA-receptors mediated [6]. Besides, cholinergic abnormalities have been observed in AD as, for instance, a reduction in acetylcholine receptor levels or dysfunctions of cholinergic signal transmission, which are of critical importance in brain areas involved in learning, memory, and emotional responses.

However, despite the extensive efforts in developing therapeutic interventions to target Aβ [7], acetylcholinesterase (AChE) [8,9,10,11], glutamatergic transmission [12] for slowing down the course of the pathology and reducing symptomatology, a decisive strategy has not yet been identified.

Given the role of Aβ in AD etiopathogenesis, there has been increasing interest in the synthesis of new molecules acting as inhibitors of Aβ aggregation and, in this context, carbazole alkaloids represent a promising class of drugs [13,14]. It is noteworthy that carbazole derivatives have been largely investigated in the last decades for their multifaceted biological properties (antibacterial, anti-inflammatory, psychotropic, anti-histamine, antitumor) [15,16,17,18,19,20,21,22,23] and, most recently, antioxidant and neuroprotective activities [11,24,25]. In particular, carbazole-containing arylcarboxamides are β-secretase (BACE1) inhibitors [26]; carbazole thiazoles are efficient, *in vitro*, in inhibiting β-amyloid formation [13], some dibenzofuran/carbazole derivatives show an improved anti-AD activity, combining the cholinesterase inhibition and anti-Aβ aggregation ability [27]. Moreover, *N*-substituted carbazoles have been reported as neuroprotective agents with potent anti-oxidative activity, showing modest to good neuroprotective effects on neuronal cells HT22 against cell injury induced by glutamate or homocysteic acid (HCA) [24]; tacrine-carbazole hybrids were developed as potential multifunctional anti-Alzheimer agents for their cholinesterase inhibitory and radical scavenging activities [11].

**Figure 1:**
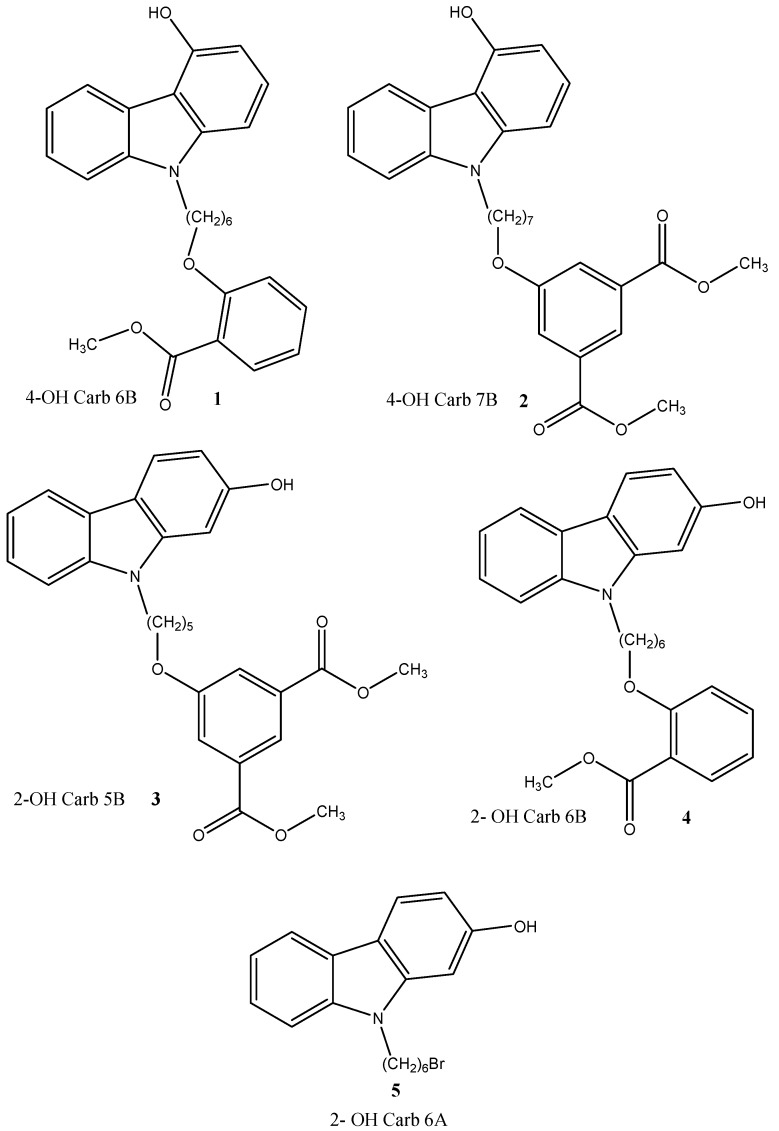
*N*-alkyl carbazole derivatives.

**Figure 2:**
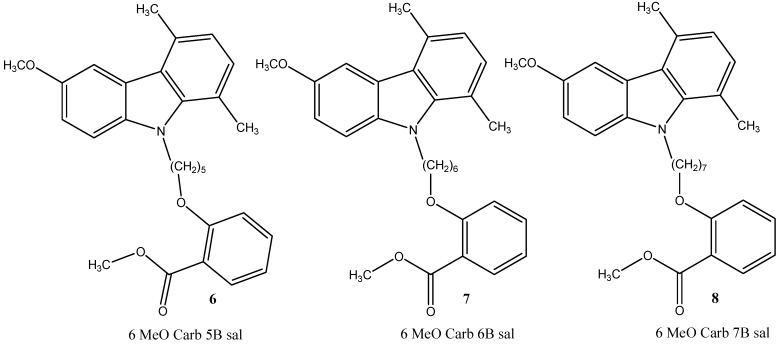
*N*-alkyl 1,4-dimethyl-carbazole derivatives.

Assuming that the reduction of insoluble Aβ aggregates may counteract the progressive degeneration occurring in AD, we have recently synthesized two series of *N*-alkyl carbazole derivatives (Figure 1 and Figure 2) [28] here investigated *in silico* and *in vitro* for their ability to promote an increase of soluble Aβ peptides.

## 2. Results and Discussion

### 2.1. Molecular Docking Simulation

We have recently synthesized two series of *N*-alkyl carbazole derivatives [28] in order to study the ability of these compounds to enhance Aβ 40 and Aβ 42 solubility. We performed preliminary docking simulations using, as a molecular target, the three dimensional NMR structure of the peptide in α helical conformation [29] (PDB code 1IYT). The two compounds 2-OH CARB 6B (**4**) and 6-MeO CARB 5B sal (**6**) display two distinct binding modes both involving key residues for fibrillization. In fact, it has been demonstrated that the intermolecular side-chain contacts responsible for fibril formations are formed between the odd-numbered residues of β strand 1 of the n^th^ molecule and the even-numbered residues of β strand 2 of the (n−1)^th^ molecule [30] (PDB code 2BEG). Particularly important in this context are the aminoacids belonging to the segment of residues Leu 17-Ala 21 and residue Asp 23 which, in the fibrillar aggregate, is involved in an intermolecular salt-bridge with Lys 28. Docking simulations demonstrated how the compound 2-OH CARB 6B (**4**) is stabilized by a π-π stacking between its methyl-benzoate ring and residue Phe 19 and by two hydrogen bonds: the first between the oxygen moiety before this ring and Asp 23, the second by the hydroxyl group and Lys 16. Moreover, further van der Waals interactions are formed by the methylen groups of the compound and residues Lys 16 and Phe 20.

**Figure 3:**
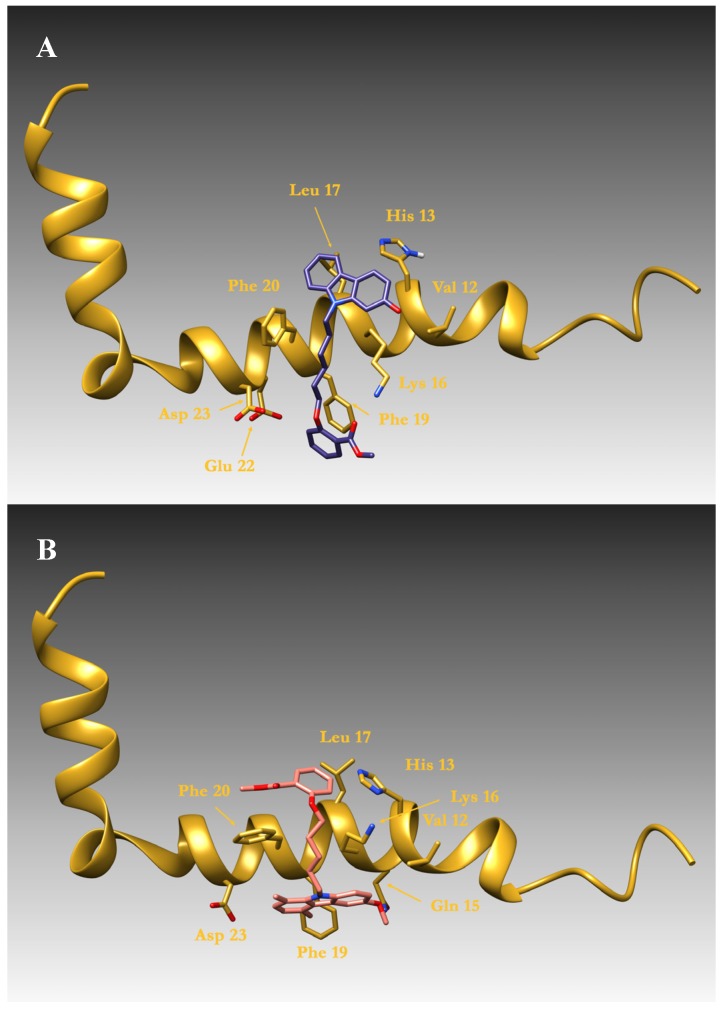
The three dimensional model of Aβ peptide is schematically reported as a gold ribbon cartoon. Residues involved in ligand binding are drawn as sticks. Panels (**A**) and (**B**) report, respectively, the binding mode of the compound 2-OH CARB 6B (**4**), drawn as violet sticks, and 6-MeO CARB 5B sal (**6**) bound to the helical peptide, drawn as pink sticks.

Finally the carbazole moiety makes van der Waals contacts with Val 12, His 13 and Leu 17 residues (Figure 3, Panel A). A head-to-tail binding mode is adopted by 6MeO CARB 5B sal (**6**). Also in this case, van der Waals interactions are formed by the methylen groups of the compound and residues Lys 16 and Phe 20, as in the compound 2-OH CARB 6B (**4**), while the carbazole moiety interacts with Phe 19 and the methoxy group interacts by an hydrogen bond with Gln 15. Additionally, the methyl-benzoate ring makes a van der Waal contact with Leu 17.

Moreover, our ligands, in both cases, protect the segment 17–21 of the Aβs peptides, increasing the solubility of the peptide and preventing the transition from the α helical peptide to a fibrillar β sheet. Summing up, docking studies evidenced that compounds **4** and **6** show two different binding modes. Regarding compound **4**, the hydroxyl group in position 2 of the carbazole moiety makes a H bridge with Lys16 whereas the oxygen on the chain is involved in another H bridge with Asp23. For compound **6** the presence of a methoxy group in position 6 of the carbazole moiety is required, which makes a H bridge with Gln15. Additionally, in both molecules, the distance between the oxygen on the chain and the N atom of carbazole scaffold play a decisive role in the interaction with Aβ peptide.

### 2.2. Biology

**Figure 4:**
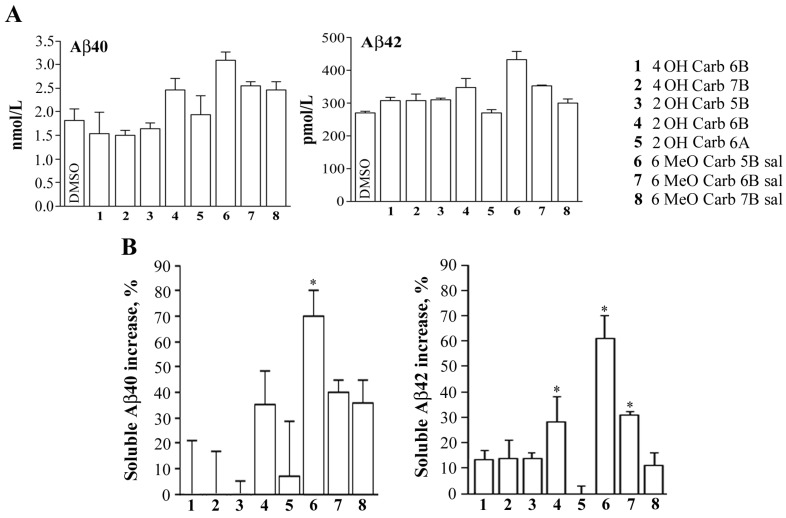
Effect of *N*-alkyl carbazole derivatives on soluble Aβ peptides in neuronal cell cultures. Conditioned media of N2a cells treated for 16 h with the indicated compounds (10 μM) were subjected to Aβ 40 and Aβ 42 specific ELISA. Results are expressed in terms of soluble Aβ concentration (**A**) and percentage increase versus DMSO-treated samples (**B**). Graphed data represent means ± S.E.M. for three independent experiments (* *p* < 0.01).

In order to verify, *in vitro*, the ability of the different *N*-alkyl carbazole derivatives (Figure 1 and Figure 2) to influence the solubility Aβ peptides, we exposed neuronal N2a cells to 10 μM of the indicated compounds dissolved in DMSO (Figure 4). Control samples received the same volume of solvent (1 μL/mL culture medium). At the end of the incubation period, the conditioned media were collected, centrifuged and subjected to Aβ sandwich ELISA specific for quantitative evaluation of soluble Aβ 40 and Aβ 42.

The results shown in Figure 4 indicate that, among the tested compounds, 2OH CARB 6B (compound **4**) and 6 MeO CARB 5B sal (compound **6**) robustly increased the concentration of soluble Aβ 40 and Aβ 42 in the cell media.

The increase in Aβ soluble forms may be due either to the interference with the amyloid aggregation process or, alternatively, with the biosynthetic pathway leading to the production of Aβ fibrils. Further studies to definitely address these issues are underway. Even though the size of aggregated β-amyloid responsible for the observed neurotoxicity and the mechanism of aggregation are not yet fully explained, the prevention of fibril formation still represents a major goal to reduce β-amyloid neurotoxicity and numerous carbazole derivatives have been synthesized as inhibitors of β-amyloid fibril formation. Besides, the synthesis of multifunctional anti-Alzheimer agents with a carbazole scaffold represents another approach with high potential for the palliative treatment of AD, for instance molecules holding high neuroprotective activity and that act either as AChE inhibitors and antioxidant agents [11,24]. In this regard, it should be highlighted that the substitution on the N atom of the carbazole moiety with a bulky group is mainly implicated in rising the neuroprotective activity of these derivatives, a feature that could be shared as well with our compounds. However, it is also true that many drugs have been proved to be very efficient *in vitro* but data coming from *in vivo* studies are few, especially because most of them are unable to efficiently pass through the blood brain barrier (BBB), which represents one of the major obstacle to the entry of adequate therapeutic concentrations of drugs into the brain. Additionally, ABC-transporters within the brain capillary endothelial cell membranes allow to remove drugs from the central nervous system limiting their uptake, as it has been reported using a fluorescent carbazole derivative [13]. Of course, these are preliminary results but may encourage further studies addressed to investigate other features of our molecules, using also an *in vivo* model such as transgenic mice overexpressing β-amyloid, which may have a potential impact for further pharmacological development in Alzheimer’s therapy.

## 3. Experimental

### 3.1. Modeling

In order to evaluate the potential binding activity of our compounds, we performed preliminary docking simulations using as molecular target the three dimensional structure of the Aβ peptide in α helical conformation as derived from NMR experiments [29] (PDB code 1IYT). Out of the 10 atomic models we chose the first (Model 1) and we further minimized it using simulated annealing procedures. The structure minimization has been performed with the program CNS 1.3 [31]. All the molecular structures of the ligands we screened “*in silico*” were built using the program “Msketch v.5.8.1” (Chemaxon Ltd., Cambridge (MA), USA) and energy minimized with Chimera [32,33] using the Amber ff99SB force field [34]. Docking simulation was performed using the program GOLD v5.2.2. The following residues on the Aβ peptide were defined with flexible side chains: His13, His14, Lys16, Leu17, Phe19, Phe20, Glu22, Asp23 allowing a free rotation of their side chains. Dockings were performed under Standard default settings mode: for each molecule tested, number of islands was 5, population size of 100, number of operations was 100,000, a niche size of 2, and a selection pressure of 1.1. The ChemPLP scoring function has been adopted.

The binding site was defined as centred around the CG atom of Phe20, with a radius of 20 Å. The top ranked molecules (mol **4** and mol **6**) shown a fitness of 95.41 and 93.51 respectively. The results obtained by these simulations allowed us to define the binding modes of the ligand tested with precision. All the figures were drawn with the program Chimera [32].

### 3.2. Cell Culture and Treatments

The cells used in this study [mouse Neuro-2a (N2a), stably expressing wild-type human APP695] were grown in 50% Dulbecco modified Eagle medium (DMEM), 50% OptiMEM with 0.1 Mm nonessential amino acids, 200 μg/mL geneticin, and 5% fetal bovine serum. The different compounds (prepared as 10 mM stock solutions in DMSO) were diluted to the final concentrations immediately before use.

### 3.3. Aβ ELISA

AβX-40 and AβX-42 ELISA kits (Wako Chemicals GmbH, Neuss, Germany) were used to determine the concentration of soluble Aβ peptides into supernatant media from cultured cells, as described previously [35]. Briefly, at the end of treatments, the conditioned media were collected, spun at 3000 g for 10 min at 4 °C to remove cell debris, and stored at −80 °C until use. Immediately after thawing, conditioned media were diluted 40 times with 1× dilution buffer and 100 μL from each sample were used. ELISA tests were carried out following the manufacturer protocols and the levels of Aβ peptides were calculated according to the standard curves prepared on the same ELISA plates.

### 3.4. Statistical Analysis

Results are expressed as means ± S.E.M. Data were analyzed by One-way ANOVA followed by Dunnett’s test.

## 4. Conclusions

Two series of compounds (Figure 1 and Figure 2) underwent molecular docking simulation and cell-based analyses in order to verify their potential interfering activity on Aβ aggregation. All together, the obtained results indicate that the two most promising compounds in docking simulations (2OH CARB 6B (**4**) and 6 MeO CARB 5B sal (**6**)) also exhibited the higher activity *in vitro*. Other studies are ongoing for a better understanding of the underlying mechanism by which the molecules act in our cell-based system. However, we believe that these observations may bring an important contribution with a potential impact on the effective antiaggregant strategy in AD.
